# Effects of low- and high-intensity physical exercise on physical and cognitive function in older persons with dementia: a randomized controlled trial

**DOI:** 10.1186/s13195-020-00597-3

**Published:** 2020-03-19

**Authors:** L. M. J. Sanders, T. Hortobágyi, E. G. A. Karssemeijer, E. A. Van der Zee, E. J. A. Scherder, M. J. G. van Heuvelen

**Affiliations:** 1 Center for Human Movement Sciences, University of Groningen, University Medical Center Groningen, Antonius Deusinglaan 1, 9713 AV Groningen, The Netherlands; 2 Department of Geriatric Medicine, Donders Institute for Brain, Cognition and Behaviour, Radboud University Medical Center, Geert Grooteplein Zuid 10, 6525 GA Nijmegen, The Netherlands; 3 Groningen Institute for Evolutionary Life Sciences (GELIFES), University of Groningen, Nijenborgh 7, 9747 AG Groningen, The Netherlands; 4 Department of Clinical Neuropsychology, VU University Amsterdam, Van der Boechorststraat 1, 1081 BT Amsterdam, The Netherlands

**Keywords:** Dementia, Cognitive decline, Physical exercise, Exercise intensity, Dose-response relationship, ApoE4

## Abstract

**Background:**

Potential moderators such as exercise intensity or apolipoprotein-E4 (ApoE4) carriership may determine the magnitude of exercise effects on physical and cognitive functions in patients with dementia (PwD). We determined the effects of a 24-week aerobic and strength training program with a low- and high-intensity phase on physical and cognitive function.

**Methods:**

In an assessor-blinded randomized trial, 91 PwD (all-cause dementia, recruited from daycare and residential care facilities, age 82.3 ± 7.0 years, 59 women, Mini-Mental State Examination 20.2 ± 4.4) were allocated to the exercise or control group. In the exercise group, PwD participated in a walking and lower limb strength training program with 12 weeks low- and 12 weeks high-intensity training offered three times/week. Attention-matched control participants performed flexibility exercises and recreational activities. We assessed adherence, compliance, and exercise intensity for each session. We assessed physical (endurance, gait speed, mobility, balance, leg strength) and cognitive (verbal memory, visual memory, executive function, inhibitory control, psychomotor speed) functions with performance-based tests at baseline and after 6, 12, 18, 24, and 36 weeks (follow-up). ApoE4 carriership was determined post-intervention.

**Results:**

Sixty-nine PwD were analyzed. Their mean attendance was ~ 60% during the study period. There were no significant effects of the exercise vs. control intervention on endurance, mobility, balance, and leg strength in favor of the exercise group (Cohen’s *d* = 0.13–0.18). Gait speed significantly improved with ~ 0.05 m/s after the high-intensity phase for exercise participants (Cohen’s *d* = 0.41) but declined at follow-up. There were no significant effects of the exercise vs. control intervention on any of the cognitive measures (Cohen’s *d* ~ − 0.04). ApoE4 carriership did not significantly moderate exercise effects on physical or cognitive function.

**Conclusions:**

Exercise was superior to control activities for gait speed in our sample of PwD. However, the training effect provided no protection for mobility loss after detraining (follow-up). There were no beneficial effects of the exercise vs. control group on cognitive function. Exercise intensity moderated the effects of exercise on gait speed. ApoE4 carriership moderated the effect of exercise on global cognition only (trend level).

**Trial registration:**

Netherlands Trial Register, NTR5035. Registered on 2 March 2015.

## Background

The number of older persons with dementia (PwD) is growing from 50 million worldwide in 2017 to 80 million in 2030 [1]. Dementia is characterized by progressive neurodegeneration and severe functional losses. The clinical relevance of pharmacological treatments remains uncertain, and the risk of adverse reactions is high [2]. Exercise may be a treatment alternative to drugs to slow functional declines in dementia. In healthy older adults, both aerobic and strength exercises are associated with improvements in cognitive functions such as executive function, inhibitory control and episodic memory [3–5], and physical functions, i.e., muscle strength, balance, functional reach, mobility, and endurance [4, 6–9]. Regrettably, the effects of exercise on these cognitive and physical functions in PwD have been inconsistent [5, 10–14]. In PwD, combined aerobic and strength exercise appears to be more effective for cognitive and physical benefits than aerobic training only [11].

Neuroprotective effects of exercise may be mediated by exercise-induced increases in brain-derived neurotrophic factor (BDNF), insulin-like growth factor-type I (IGF-1), vascular endothelial growth factor (VEGF), and homocysteine [15–23] thereby promoting structural and connectivity changes in the brain areas important for memory and executive function, e.g., frontal and temporal lobes and hippocampus [24–27].

There is no conclusive evidence for exercise as a treatment modality for PwD. Identifying the variables that moderate the relationship between cognition and physical function is needed to optimize exercise programs [28]. A few potential moderators have been identified. For example, the presence of the apolipoprotein-E4 (ApoE4) allele, a risk factor for Alzheimer’s disease (AD) [29], may mediate the magnitude of exercise effects. Accumulation of neuronal and physiological damage in ApoE4 carriers may negate the beneficial effects of physical activity [30, 31]. Conversely, ApoE4 carriers may be more responsive to exercise [32], perhaps because lower functional levels at baseline [33–36] leave more room for improvement. In addition to ApoE4 carriership, exercise intensity may determine the magnitude of exercise effects. Exercise-induced changes in the aforementioned neurobiological factors may be dose-dependent, as evidenced by studies in rodents [37, 38] and humans [18, 39, 40]. Furthermore, exercising at moderate-to-vigorous intensities is recommended over lighter intensities for cardiovascular, muscular, and neuromotor benefits in healthy young and old adults [41]. Whether this is true also for cognitive functions is undetermined [5].

In the current sample of PwD, we aimed to determine (1) the feasibility of low- vs. high-intensity combined aerobic and strength training, (2) the dose-response effects of low- and high-intensity combined aerobic and strength exercise on physical and cognitive functions, (3) if high- vs. low-intensity exercise has differential effects on physical and cognitive functions, and (4) whether ApoE4 moderates the effects of exercise. We hypothesized that (1) a 6-month combined aerobic and strength training program with a low- vs. high-intensity phase would be feasible in our sample, (2) the exercise program would reduce the rate of decline in physical and cognitive function, (3) the beneficial effects would be greater after high- vs. low-intensity exercise, and (4) that ApoE4 carriership would moderate the effects of exercise on physical and cognitive functions.

## Methods

### Design

We assessed the effects of a 6-month combined aerobic and strength training program with a low (LI, week 1–12) and high (HI, week 13–24) intensity phase compared to a control program of matched attention in a randomized controlled study design. We performed blinded assessments of cognitive and physical functions at T0, T12 (after 12 weeks), and T24 (after 24 weeks). Brief (blinded) assessments of a selection of cognitive and physical functions were performed at T6 (after 6 weeks), T18 (after 18 weeks), and T36 (follow-up after 36 weeks). After 24 weeks, a saliva sample was taken to determine ApoE4 carriership. We included patients with mild-to-moderate dementia who attended daycare or resided in residential care facilities with open front door policies. A power analysis on our design using a small-to-medium effect size (ES), alpha = 5%, power = 80%, and expected dropout of 25% resulted in a minimal sample size of 59 participants per group. The ES for this power analysis was based on the eight most sensitive tests in a previous study by Bossers et al. [11] (ES = 0.21–0.31) with correlations between pretest and post-test of *r* = 0.65–0.85.

### Participants

Between September 2015 and October 2017, participants were recruited from 13 health care locations that provided daycare or residential care for PwD. Health care staff selected potential participants based on the instructions from the researchers. These instructions were that potential participants had to be able to walk with or without an assistive walking device had to have sufficient ability to follow instructions and had to be interested in participating in a scientific study. With oral consent from participants and their caregivers, health care staff provided the names and contact information of the selected potential participants. The researchers provided these potential participants and their caregivers with further oral and written information. After oral and written informed consent was obtained from participants and their caregivers, participants were screened further for eligibility by a trained research assistant. Participants were then included if they met the following criteria: a dementia diagnosis determined by a primary care physician or geriatrician (the Dutch College of General Practitioners advises to use the Diagnostic and Statistical Manual of Mental Disorders fourth edition (DSM-IV) for the diagnosis of dementia [42]), age ≥ 65 years, a physician-determined all-cause dementia diagnosis, able to complete the Timed Up & Go (TUG [43]) with or without an assistive device, and a Mini-Mental State Examination (MMSE [44]) score > 10 corresponding to mild-to-moderate dementia. Participants were excluded if they met one of the following criteria: wheelchair-bound; presence of severe cardiovascular problems that limit physical activity or brain trauma, epilepsy, progressive or terminal disease, and/or depression; history of alcoholism and/or Korsakoff’s syndrome; severe visual or auditory problems; non-fluent in the Dutch language; and mental incompetence without a legal guardian.

### Procedures

The Ethical Committee of the University Medical Center Groningen approved the study (METc 2014/523). The Dutch Trial Registration number is NTR5035. We obtained oral and written informed consent from participants and their caregivers. The study was conducted in accordance with the Declaration of Helsinki (64th amendment).

Participants were randomly assigned to the combined aerobic and strength training intervention (“exercise”) or control intervention (“control”) with an allocation ratio of 1:1. We stratified participants according to MMSE, gender, and health care location, so that the number of exercise vs. control participants was approximately equally distributed according to MMSE score, gender, and health care location.

The program duration was 24 weeks for both the exercise and control interventions. Participants in the exercise and control interventions were offered 72 individualized sessions (3/week for 24 weeks—for the exercise group, this amounted to 36 walking sessions and 36 strength exercise sessions) of 30 min each. This combination of combined walking and strength exercise, 3 sessions/week for 24 weeks, previously showed the highest efficacy on physical and cognitive outcomes in PwD [11, 45]. Each session was supervised on a one-on-one basis by a trained research assistant who was assigned to the participant. Each research assistant kept a log of each session. The log was used to continuously record the heart rate and rate of perceived exertion (RPE) during the session, activity specifics, participant satisfaction, and noteworthy details. The research assistants used these variables to establish the targeted exercise intensity. In addition, the research assistants evaluated the quality of every strength exercise according to the protocol (such as exercise execution, posture, leg straightening) on a scale of 1 (insufficient) to 4 (good).

### Exercise intervention

#### Aerobic sessions

The aerobic sessions consisted of outdoor walking. If the weather did not allow for outdoor walking or the participant rejected outdoor walking, walking was performed indoors.

Subjects in the exercise intervention exercised at LI for the first 12 weeks and at HI for the subsequent 12 weeks. The target intensity of sessions was determined in correspondence with the American College of Sports Medicine (ACSM [46]) guidelines for “low” and “moderate-to-high” intensity exercise. The intensity of the aerobic sessions was monitored objectively every 5 min using a MIO Link Continuous Heart Rate Wrist Band. Subsequently, training intensity was determined objectively using the percentage of maximum heart rate (%HRmax, with HRmax = 208 − (0.7 × age)) and subjectively with observer-determined RPE using a Borg scale. The Borg scale ranges from 6 to 20, with 6 corresponding to minimal intensity and 20 to maximal intensity. In the LI phase, the target RPE was 9–11 and target HR was 57–63%HRmax. In the HI phase, participants performed interval training with alternating 4-min peak performance at RPE 15–16 and 83–89%HRmax and 3-min active rest at RPE 13–14 and 71–77%HRmax. Although we do not deem the observer-rated RPE to be superior to HR measures when determining exercise intensity, we instructed the research assistants to rely on the observer-rated RPE in case of discrepancies between RPE and heart rate which could be due to beta blockers. Walking intensity could be increased or decreased by adapting walking speed and the number of passive or active rests.

#### Strength sessions

Lower limb strength exercises can help enhance walking ability and produce a stronger neuromotor stimulus [11]. Four lower limb exercises were performed during the strength sessions in a fixed sequence: (1) knee extension while sitting, (2) plantar flexion (toe standing), (3) hip abduction (side leg lifts), and (4) hip extension (back leg lifts). A chair was used for support. Per session, the muscle contractions were either isometric, concentric, or eccentric (so that 12 isometric, 12 concentric, and 12 eccentric contraction sessions were offered throughout the exercise intervention). We used only the target RPE to determine the intensity because no significant increases in heart rate were expected.

The intensity of the strength sessions was determined subjectively with the observer-determined RPE. In the LI phase, the target RPE was 9–11. In the HI phase, the RPE was 13–16. Exercise intensity could be increased or decreased by adapting the number of sets and repetitions (Additional file 1: Appendix 1). Ankle weights were added in the HI phase per 0.5 kg for all exercises except toe stands. The added weight was increased equally for all exercises (except toe stands).

### Control intervention

The control intervention consisted of flexibility exercises and recreational activities (matched attention). The flexibility exercises included upper and lower body exercises such as neck or shoulder rotation and stretching knee flexors and extensors. No weights were used. Additionally, recreational activities such as board games or social visits were performed depending on the participants’ preference.

### Measurements

#### Medical information

We collected information on dementia diagnosis [42], comorbidities (Functional Comorbidity Index-18 (FCI-18 [47]), and medication use from medical files kept by each participants’ general practitioner. Anticholinergic and sedative drug burden was represented by the Drug Burden Index (DBI [48]).

#### ApoE4 status

We used sterile buccal swabs to take saliva samples for APOE genotyping. Buccal samples were analyzed using the real-time polymerase chain reaction (PCR) method [49]. This resulted in six different potential APOE genotypes (e2/e2, e2/e3, e2/e4, e3/e3, e3/e4, e4/e4).

#### Physical function

We used five physical function tests that are deemed suitable for PwD [50]. Additional file 1: Appendix 2a describes these tests in more detail. The 6-minute walk test (6MWT) [51] measures endurance. The Short Physical Performance Battery (SPPB) [52] assesses lower body strength and functional mobility. We measured habitual gait speed with the 6-meter walking speed test (6MWS). We used the FICSIT-4 [53] as a static balance measure. We assessed lower body muscle strength with the Quadriso table (see Additional file 1: Appendix 2a for details). The Quadriso table is a lower body force-measuring device based on the Quadrisotester of Verkerke et al. [54]. The TUG measures functional mobility.

All tests were performed at T0, T12, and T24. 6MWS and leg strength were assessed at T6, T18, and T36 as well.

#### Cognitive function

We assessed cognitive function with neuropsychological tests that were previously used in PwD [50]. Additional file 1: Appendix 2b describes these tests. Global cognition was assessed with the MMSE. We measured the psychomotor speed with the Trail Making Test A (TMTA) [55]. The Digit Span Forward (DSFW) and Backward (DSBW) [56] measure verbal memory span and verbal working memory, respectively. The Visual Memory Span Forward and Backward (VMSFW and VMSBW) [56] are measures of respectively visual memory span and visual working memory. The STROOP test [57] is used to assess basic attentional processing and inhibitory control. We used the phonemic fluency test (Fluency) [58] as an executive function measure.

All tests were performed at T0, T12, and T24. The STROOP test was also performed at T6, T18, and T36.

### Statistical analyses

We used SPSS 25.0 (IBM: Armonk, NY) to compute means and standard deviations and to analyze the data with a two-tailed significance set at *p* < 0.05. Scores on the TMTA, STROOP interference, TUG, and 6MWS were right-skewed and therefore natural-log transformed. We accounted for missing values on cognitive and physical variables at T0, T6, T12, T18, T24, and T36 with multiple imputation (9.2% of the cognitive variables missing (3.2% T0, 5.3% T6, 10.0% T12, 6.8% T18, 12.9% T24, and 20.3% T36) and 9.2% of the physical variables missing (2.4% T0, 13.0% T6, 8.9% T12, 10.1% T18, 11.1% T24, and 19.6% T36)); automatic model setting; 40 imputations; and 100 iterations (done separately for physical vs. cognitive variables and exercise vs. control group). Reasons for missing values were illness, refusal of assessment, or being otherwise (temporarily) unavailable. We performed adapted intention-to-treat analyses by selecting all individuals who completed ≥ 5 assessments independent of attendance (*N* = 69, 39 exercise group). Group differences for physical and cognitive outcomes were assessed with analyses of covariance (ANCOVA) with continuous baseline variables as covariates.

To determine the magnitude of exercise effects, we calculated Cohen’s *d* effect sizes (ESs) using the following formula:

$$ d=\frac{\left({\mathrm{post}}_{\mathrm{exp}}-{\mathrm{pre}}_{\mathrm{exp}}\right)-\left({\mathrm{post}}_{\mathrm{cont}}-{\mathrm{pre}}_{\mathrm{cont}}\right)}{\sqrt{\frac{\frac{s_{\mathrm{pre},\exp}^2\times {n}_{\mathrm{exp}}+{s}_{\mathrm{pre},\mathrm{cont}}^2\times {n}_{\mathrm{cont}}}{n_{\mathrm{exp}}+{n}_{\mathrm{cont}}}+\frac{s_{\mathrm{post},\exp}^2\times {n}_{\mathrm{exp}}+{s}_{\mathrm{post},\mathrm{cont}}^2\times {n}_{\mathrm{cont}}}{n_{\mathrm{exp}}+{n}_{\mathrm{cont}}}}{2}}} $$

where “post” represents T12 or T24 measurements, “exp” represents exercise, and “cont” represents the control group. Values of *d* = 0.20, *d* = 0.50, and *d* = 0.80 indicate small, medium, and large effect sizes [59], respectively; 95% confidence intervals (CIs) for *d* were calculated using the formula *d* ± 1.96 × SE, with [60]:

$$ \mathrm{SE}=\sqrt{\left(\frac{n_{\mathrm{exp}}+{n}_{\mathrm{cont}}-1}{n_{\mathrm{exp}}+{n}_{\mathrm{cont}}-3}\right)\times \left(\left(\frac{4}{n_{\mathrm{exp}}+{n}_{\mathrm{cont}}}\right)\times \left(1+\frac{d^2}{8}\right)\right)} $$

We considered an effect to be a dose-response effect with respect to intensity if the change from baseline T24 (LI+HI phase) was higher or equal (as we expected that potential beneficial effects would become less pronounced over the course of the study) to the change from baseline T12 (LI phase). We used the results of the ANCOVAs (as previously described) as well as the qualitative comparison of ESs to compare the effects after the LI phase vs. the full study period (LI+HI phase).

To examine ApoE4 as a potential moderator, we conducted a repeated measures ANOVA with physical and cognitive outcome variables as dependent variables, time of measurement (baseline and T24) as a within-subject factor, and group (exercise vs. control) and carrier (ApoE4 carrier vs. non-carrier) as between-subject factors. We considered ApoE4 to moderate the effects of exercise on physical or cognitive functions if there was a significant three-way Group*Carrier*Time interaction.

## Results

**Fig. 1:**
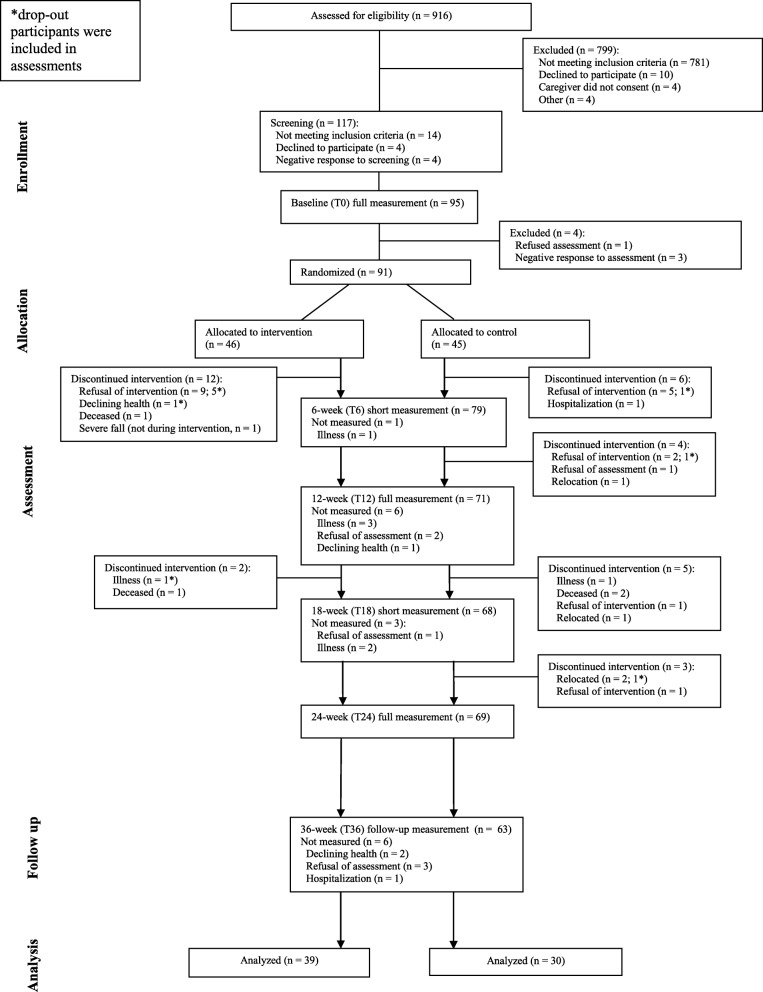
CONSORT flowchart

Figure 1 shows the flowchart of the study. Of the 916 persons that were screened for eligibility, 91 were randomized (*N* = 46 exercise vs. *N* = 45 control; mean age = 82.3 ± 6.96; 59 women; a median level of education = secondary lower education; mean MMSE = 20.2 ± 4.40; use of walking aid *N* = 50). Of these 91 participants, 22 (24%) participants dropped out after allocation. There were significantly more dropouts in the control (*N* = 15 (33%)) vs. exercise (*N* = 7 (15%)) intervention (*χ*^2^(1) = 4.08, *p* < 0.05). There were no differences with respect to age, gender, level of education, and baseline MMSE between participants who dropped out vs. participants who remained in the study (*N* = 69). Figure 1 shows the time and reasons for dropout.

### Intention-to-treat analyses

The current analyses involve the participants who performed ≥ 5 assessments (*N* = 69; *N* = 39 exercise vs. *N* = 30 control; mean MMSE = 20.6 ± 4.38; 43 women). Table 1 shows the baseline characteristics of this sample.

**Table 1:** Sample characteristics at baseline

Characteristic	Exercise (*N* = 39)	Control (*N* = 30)
Age (mean, SD)	81.7 (7.16)	82.1 (7.51)
Gender (*N* women, % total)	21 (53.8)	22 (73.3)^†i^
Level of education (*N*, % total)		
1 = primary education only	10 (25.6)	8 (26.7)
2 = secondary lower education	25 (64.1)	19 (63.3)
3 = secondary higher education	4 (10.3)	3 (10.0)
Use of walking aid at baseline (*N*, % total)	17 (43.6)	19 (63.3)
Dementia diagnosis according to medical file^a^ (*N*, % total)		
1 = Alzheimer’s disease (AD)	14 (35.9)	7 (23.3)
2 = vascular dementia (VD)	3 (7.7)	1 (3.3)
3 = mixed (AD+VD)	3 (7.7)	5 (16.7)
4 = dementia with Lewy bodies (DLB)	0 (0.0)	1 (3.3)
5 = others/unspecified^b^	11 (28.2)	12 (40.0)
MMSE^c^ (mean, SD)	21.4 (3.94)	19.5 (4.77)^†j^
APOE^d^ genotype (*N*, % total)		
Carrier (e3/e4 and e4/e4)	18 (46.2)	12 (40.0)
Non-carrier (e2/e2, e2/e3, e3/e3)	21 (53.8)	18 (60.0)
Number of medications used^e^ (mean, SD)	5.2 (2.45)	5.1 (2.74)
Use of beta blockers (*N*, % total)	21 (53.8)	14 (46.7)
DBI^f^ (mean, SD)	0.24 (0.38)	0.22 (0.31)
FCI^g^ (mean, SD)	2.4 (1.66)	2.7 (1.96)
BMI^h^ (mean, SD)	27.3 (3.53)	27.6 (3.71)

^a^*N* = 12 missing; ^b^diagnosis of “dementia” or “dementia syndrome”; ^c^Mini-Mental State Examination; ^d^apolipoprotein E, within the carrier group *N* = 2 homozygote in exercise group, *N* = 1 homozygote in the control group; ^e^*N* = 1 missing; ^f^Drug Burden Index, *N* = 4 missing; ^g^Functional Comorbidity Index, *N* = 9 missing; ^h^body mass index. ^†^Significant at *p* < 0.01. ^i^*χ* (1) = 2.74, *p* = 0.098; ^j^*F*(1, 67) = 3.03, *p* = 0.086

Additional file 1: Appendix 3 presents training characteristics for the exercise (LI vs. HI phase) and control groups. The overall attendance was ~ 60% for the exercise group and ~ 70% for the control group. Attendance was not significantly different for the walking vs. strength sessions, LI vs. HI phase and exercise vs. control group. Participant satisfaction was generally high but lowered for the HI vs. LI walking sessions. For the HI vs. LI strength sessions, the RPE and number of repetitions were significantly higher with the added weight being ~ 0.71 kg (there were no added weights in the LI sessions). There was no loss of quality for the HI vs. LI strength exercises. The contrast between LI and HI walking was less pronounced. The total distance walked in 30 min was ~ 40 m higher in the HI phase (1.30 km LI vs. 1.34 km HI). However, the mean and maximum heart rates were not significantly different between LI and HI walking sessions. Furthermore, there were no significant differences in the maximum heart rate between participants with and without beta blockers. Also, there were no differences in the observer-rated RPE (for the LI walking, HI walking, LI strength or HI strength sessions) for participants with vs. without beta blockers (data not shown). The mean HR of ~ 95 b/min^−1^ during LI and HI walking sessions falls within the low to low-to-moderate intensity range, and the maximum HR of ~ 135 b/min^−1^ during LI and HI walking sessions can be considered high intensity according to ACSM guidelines [46] (given the mean age = 81.8, HRmax = 208–0.7 × 81.8 = ~ 151).

**Fig. 2:**
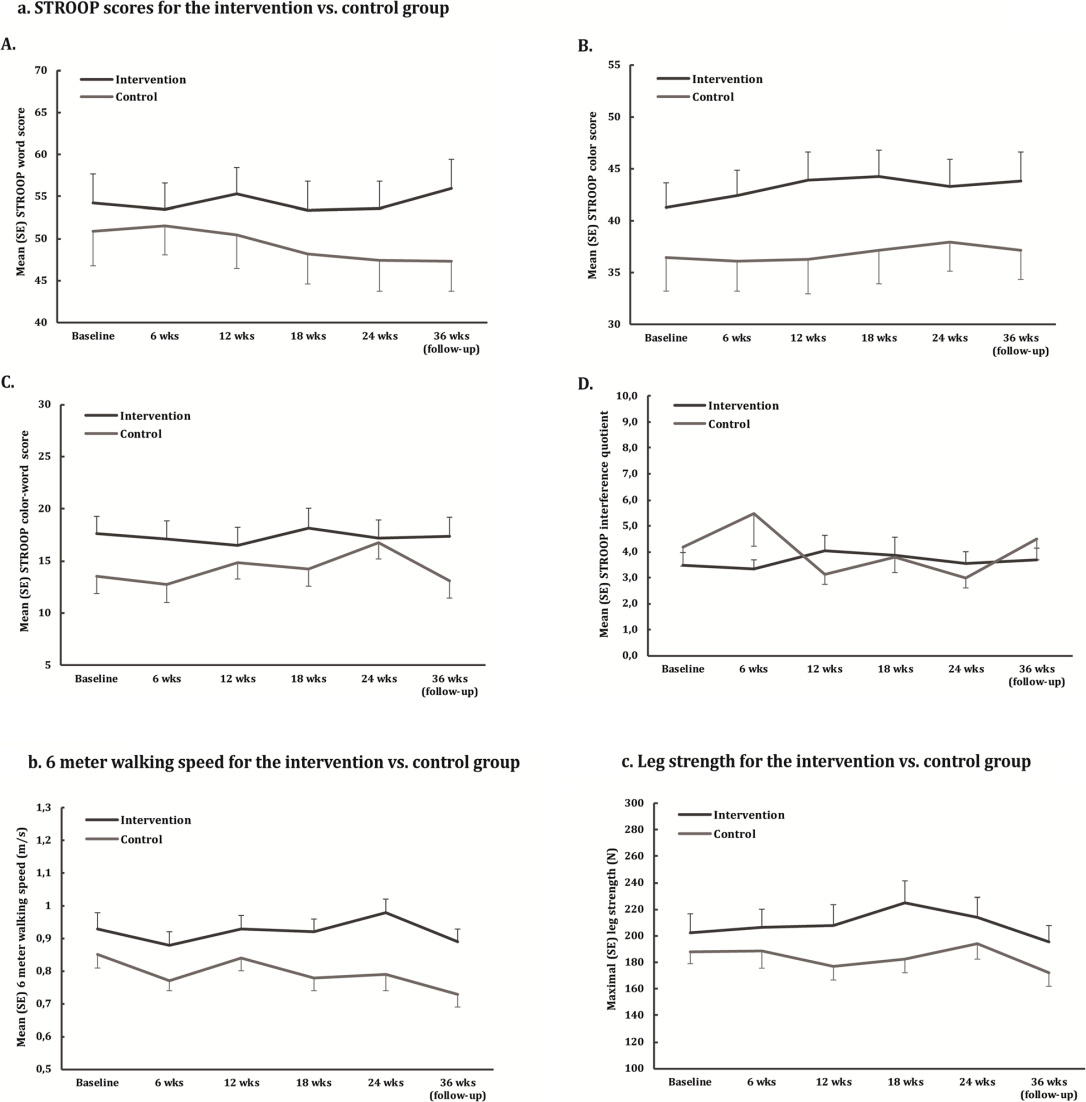
Scores on STROOP, 6-meter walking speed and leg strength for the intervention vs. control group

The exercise intervention had a significant positive effect on 6MWS after 18 (*F*(1, 66) = 5.12, *p* < 0.05) and 24 weeks (Table 2) (Fig. 2b), also after multiple testing correction at 24 weeks (alpha-correction of 0.05/17 (17 functional measurements)). The ES increased from *d* = 0.04 at T12 to *d* = 0.41 at T24 (Table 2). At follow-up, 6MWS declined and was no longer significantly higher for the exercise vs. control group (Fig. 2b) (Additional file 1: Appendix 5). There were no significant effects of the exercise vs. control intervention on the other physical measures (mean *d* = 0.18 for the LI phase and mean *d* = 0.13 for the HI phase; Table 2, Fig. 2c for leg strength).

**Table 2:** Descriptives, effect sizes, and results of ANCOVA for physical test scores

Test^a^	Group	Baseline	12 weeks	24 weeks	Effect size, baseline-12 weeks^b^	*F*(1, 66)^c^, *p*	Effect size, baseline-24 weeks^b^	*F*(1, 66)^c^, *p*
6MWT (m)	Exercise	278 (89.4)	280 (87.0)	289 (95.0)	0.18 [− 0.30, 0.65]	2.73, *p* > 0.05	0.08 [− 0.40, 0.56]	1.36, *p* > 0.05
	Control	234 (88.6)	222 (98.8)	238 (87.4)				
SPPB (score)	Exercise	8.75 (2.25)	9.19 (2.37)	8.96 (2.31)	0.28 [− 0.20, 0.76]	3.27^c^, *p* > 0.05	0.16 [− 0.32, 0.64]	2.46, *p* > 0.05
	Control	7.77 (2.08)	7.58 (2.14)	7.61 (2.41)				
6MWS (m/s)	Exercise	0.93 (0.31)	0.93 (0.25)	0.98 (0.25)	0.04 [− 0.44, 0.52]	1.46, *p* > 0.05	0.41 [− 0.07, 0.90]	12.83, *p* < 0.001**
	Control	0.85 (0.22)	0.84 (0.22)	0.79 (0.27)				
FICSIT-4 (score)	Exercise	3.36 (1.06)	3.45 (1.19)	3.30 (1.31)	0.15 [− 0.33, 0.63]	1.45, *p* > 0.05	− 0.15 [0.63, 0.33]	0.09, *p* > 0.05
	Control	2.90 (1.40)	2.81 (1.24)	3.03 (1.35)				
TUG (s)	Exercise	14.4 (6.24)	13.6 (5.56)	14.1 (6.62)	0.23 [− 0.26, 0.71]	2.35, *p* > 0.05	0.17 [− 0.31, 0.66]	1.43, *p* > 0.05
	Control	17.3 (5.56)	17.8 (7.57)	18.0 (7.20)				
Leg strength (*N*)	Exercise	202 (91.4)	208 (98.4)	214 (95.8)	0.21 [− 0.27, 0.69]	2.01, *p* > 0.05	0.07 [− 0.41, 0.55]	0.39, *p* > 0.05
	Control	188 (51.4)	177 (58.5)	194 (67.0)				

Values are mean (SD). *N* total = 69; *N* = 39 exercise vs. *N* = 30 control. Baseline-12 weeks is the low-intensity phase; baseline-24 weeks is the full study period (low-intensity and high-intensity phase). ^a^*6MWT* 6-meter walk test, *SPPB* short physical performance Battery, *6MWS* 6-meter walk speed; *TUG* Timed Up & Go. ^b^Cohen’s *d* with 95% CI, positive effect sizes are in favor of exercise group. ^c^ANCOVA with baseline as a covariate and use of walking aid as a factor, the main effect of group (exercise vs. control). **Significant at *p* < 0.001

There were no significant effects of the exercise vs. control intervention on any of the cognitive measures (mean *d* = − 0.03 for the LI phase and mean *d* = − 0.04 for the HI phase; Table 3, Fig. 2a for all STROOP scores). Both the exercise and control participants remained stable over the course of the study. There was a significant effect on the STROOP interference quotient in favor of controls after 12 weeks (Table 3), but this effect did not survive multiple testing correction (alpha-correction of 0.05/17 (17 functional measurements)).

**Table 3:** Descriptives, effect sizes, and results of ANCOVA for cognitive test scores

Test^a^	Group	Baseline	12 weeks	24 weeks	Effect size^b^, baseline-12 weeks	*F*(1, 66)^d^, *p*	Effect size^b^, baseline-24 weeks	*F*(1, 66)^d^, *p*
MMSE (score)	Exercise	21.4 (3.94)	21.0 (4.38)	20.4 (4.77)	− 0.05 [− 0.53, 0.43]	0.11, *p* > 0.05	− 0.04 [− 0.52, 0.44]	0.04, *p* > 0.05
	Control	19.5 (4.77)	19.4 (5.64)	18.8 (5.88)				
TMTA (s)	Exercise	121 (64.2)	123 (63.7)	126 (65.3)	− 0.03 [− 0.51, 0.45]	0.51, *p* > 0.05	− 0.14 [− 0.62, 0.34]	0.52, *p* > 0.05
	Control	156 (65.2)	156 (61.1)	153 (56.6)				
STROOP word (no. of correct responses)	Exercise	54.3 (21.1)	55.3 (19.7)	53.5 (20.3)	0.07 [− 0.41, 0.55]	0.64, *p* > 0.05	0.13 [− 0.35, 0.61]	1.35, *p* > 0.05
	Control	50.9 (22.5)	50.5 (22.0)	47.4 (20.1)				
STROOP color (no. of correct responses)	Exercise	41.3 (14.8)	43.9 (17.0)	43.3 (16.3)	0.17 [− 0.31, 0.65]	1.93, *p* > 0.05	0.03 [− 0.45, 0.51]	0.47, *p* > 0.05
	Control	36.4 (17.6)	36.3 (18.0)	38.0 (15.4)				
STROOP color-word (no. of correct responses)	Exercise	17.6 (10.4)	16.5 (10.8)	17.2 (10.7)	− 0.24 [− 0.72, 0.24]	0.45, *p* > 0.05	− 0.37 [− 0.85, 0.12]	0.61, *p* > 0.05
	Control	13.5 (9.05)	14.9 (8.63)	16.8 (8.83)				
STROOP interference quotient	Exercise	3.46 (3.18)	4.04 (3.68)	3.56 (2.81)	− 0.49 [− 0.98, 0.00]	4.29, *p* = 0.04*	− 0.42 [− 0.90, 0.07]	2.13, *p* > 0.05
	Control	4.19 (3.99)	3.14 (2.21)	3.00 (2.21)				
DSFW (no. of correct responses)	Exercise	6.79 (1.77)	7.11 (2.14)	6.67 (1.80)	0.15 [− 0.33, 0.63]	0.96, *p* > 0.05	− 0.13 [− 0.61, 0.35]	0.40, *p* > 0.05
	Control	6.53 (1.55)	6.58 (1.67)	6.64 (1.91)				
DSBW (no. of correct responses)	Exercise	4.00 (1.39)	4.00 (1.54)	4.01 (1.31)	− 0.03 [− 0.51, 0.45]	0.20, *p* > 0.05	0.10 [− 0.38, 0.58]	0.15, *p* > 0.05
	Control	4.17 (1.32)	4.21 (1.48)	4.04 (1.60)				
VMSFW (no. of correct responses)	Exercise	5.78 (1.95)	5.50 (1.57)	5.23 (1.50)	0.04 [− 0.43, 0.52]	2.01, *p* > 0.05	− 0.05 [− 0.53, 0.43]	1.20, *p* > 0.05
	Control	4.96 (1.80)	4.60 (1.82)	4.50 (2.05)				
VMSBW (no. of correct responses)	Exercise	4.31 (2.05)	4.23 (1.93)	4.56 (1.71)	0.14 [− 0.34, 0.62]	0.71, *p* > 0.05	0.33 [− 0.16, 0.81]	2.92, *p* > 0.05
	Control	4.30 (1.86)	3.94 (1.94)	3.93 (1.98)				
Fluency (no. of correct responses)	Exercise	18.9 (7.73)	18.3 (8.27)	21.6 (8.49)	− 0.06 [− 0.54, 0.42]	0.03, *p* > 0.05	0.13 [− 0.35, 0.61]	2.00, *p* > 0.05
	Control	14.7 (9.39)	14.6 (9.95)	16.3 (9.52)				

Values are mean (SD). *N* total = 69; *N* = 39 exercise vs. *N* = 30 control. Baseline-12 weeks is the low-intensity phase; baseline-24 weeks is the full study period (low-intensity and high-intensity phase). ^a^*MMSE* Mini-Mental State Examination, *TMTA* Trail Making Test A, *DSFW* Digit Span Forward, *DSBW* Digit Span Backward, *VMSFW* Visual Memory Span Forward, *VMSBW* Visual Memory Span Backward, *Fluency* phonemic fluency test. ^b^Cohen’s *d* with 95% CI, positive effect sizes are in favor of exercise group. ^d^ANCOVA with baseline as a covariate, the main effect of group (exercise vs. control). *Significant at *p* < 0.05

### ApoE4 moderation

ApoE4 carriers (*n* = 30) were ~ 3 years younger than non-carriers (*n* = 39) (non-significant difference) and used more beta blockers (66.7% of carriers and 38.5% of non-carries used beta blockers, *χ*^2^(1) = 5.40, *p* < 0.05). There were no other significant baseline differences, also with respect to the physical and cognitive function tests. There were no significant three-way Time*Group*Carrier interactions for any of the cognitive or physical measures (all *p* > 0.05, Additional file 1: Appendix 4a-4c).

## Discussion

### Summary of results

This is the first assessor-blinded RCT investigating the effects of LI vs. HI combined aerobic and strength exercise in PwD. Gait speed significantly improved for the exercise vs. control group after 24 weeks (*d* = 0.41, *p* < 0.05) but declined at follow-up. We found no significant effects of exercise on the other physical functions. There were no differences between the LI (mean *d* = 0.18) and HI (mean *d* = 0.13) phase. There were no effects of exercise on cognitive functions, and no differences between the LI (mean *d* = − 0.03) and HI (mean *d* = − 0.04) phase. ApoE4 carriership did not moderate the effects of exercise on physical or cognitive function.

### Feasibility of the exercise program

This exercise program was feasible in this sample of PwD. The mean attendance rate was ~ 60% in the LI and HI phase. All exercise participants were able to perform the strength exercises with and without weights. There were no serious study-related adverse events. Notwithstanding the individual supervision, the mean attendance rate was lower than what is considered necessary for functional improvements (i.e., ≥ 3 performed sessions per week) [41]. Low attendance rates were often caused by the unavailability of the participant due to illness or scheduling conflicts. Furthermore, some participants were unwilling to participate in all exercise sessions. The quality of the strength exercises was rated ~ 2.5 on average which amounts to a sufficient execution. Problems with the execution of exercises may arise due to poor physical fitness and knee or hip complaints in PwD. Perhaps a higher quality of execution would attribute to better exercise effects on functional outcomes. However, additional analyses (data not shown) did not show that higher attendance or better quality was predictive of better physical or cognitive effects in PwD.

We aimed to contrast LI with HI exercise. Overall, our results confirm the contrast between LI and HI exercise (Additional file 1: Appendix 3). However, this contrast was more pronounced for the LI vs. HI strength exercises than for LI vs. HI walking. With respect to the strength exercises, the average added weight of ~ 0.7 kg can be considered relevant given our participants’ poor physical fitness. Furthermore, the number of repetitions was higher in the HI vs. the LI strength sessions. With respect to walking, the RPE was significantly higher in the HI phase but the heart rate was not. With respect to the RPE, we chose proxy report over self-report because our pilot data (unpublished) showed that our participants had difficulties in understanding the Borg scale. We chose to continuously monitor the heart rate to have an objective measure of exercise intensity. However, we are unsure if heart rate is a reliable indicator of exercise intensity in PwD. All types of dementia are associated with dysfunction of the autonomic nervous system including heart rate variability [61]. This may influence the heart rate response to exercise in PwD. Future studies are needed to investigate whether there are differences in heart rate response to exercise in PwD vs. healthy older adults.

We had selective dropout in our sample as our baseline sample (*N* = 91) showed no differences in baseline characteristics (age, gender, education, MMSE, endurance capacity, and use of walking aid; data not shown) whereas in our analyzed sample (*N* = 69), exercise participants had higher levels of physical and cognitive functions at baseline compared with control participants. Despite starting with LI exercise, lower functioning individuals were more likely to drop out of the exercise group often within the first weeks of the study. This could perhaps have been prevented with a more gradual increase in session duration or frequency. Conversely, higher functioning individuals were more likely to drop out of the control group. This could perhaps have been prevented with more challenging control activities or better management of potential participants’ expectations for the control group. The higher dropout rate in the control vs. exercise group may be due to lower participant interest in the control vs. exercise group, perhaps because control group activities (e.g., recreational activities) were also offered at daycare facilities as part of usual care.

### Effects of exercise on physical function

Gait speed significantly improved with ~ 5% after 24 weeks. Gait speed is an important clinical measure in older adults because it is associated with the rate of cognitive decline [62], vulnerability to adverse events [63], and survival [64]. The change in gait speed for participants in the exercise group between baseline and T24 was ~ 0.05 m/s which is considered functionally meaningful [65]. Gait speed may have been the most sensitive to changes considering the nature of our exercise program, in line with the specificity principle (i.e., adaptations in gait speed are more likely after a walking program compared to adaptions in balance or sit-to-stand measures) and previous meta-analytic results that showed that gait speed was especially sensitive to progressive resistance training with higher intensities [66]. It is unlikely that the effects of exercise on gait speed were random outliers as gait speed improved in respectively 38% vs. 13% of exercise vs. control participants (change ≥ 0.05 m/s). The finding that gait speed improved more in the HI vs. the LI phase may be indicative of a dose-response effect for intensity. The LI vs. HI contrast was most pronounced for the strength sessions. These results attest to a relationship between gait speed improvements and strength improvements [67]. Although there were no significant exercise effects on leg strength, we deem a true lack of exercise effects on leg strength unlikely given previous data [11, 14, 45]. Therefore, we believe that a lack of significant (dose-response) improvements in leg strength may have resulted from our assessment method: PwD may be hesitant to generate maximum force either in fear of pain or injury, or lack of motivation. Also, PwD may have trouble comprehending the test instructions. Exercise did not provide a protective effect against gait speed losses when exercise was withdrawn, as indicated by a decline in gait speed after detraining (at follow-up), although it should be noted that the overall decline in gait speed from baseline to follow-up was smaller in the exercise group (~ 0.04 m/s) as compared to the control group (~ 0.12 m/s). The detrimental effects of detraining on physical function are well known in older persons with and without dementia [11, 68–70]. Thus, our results support the recommendation of continuous physical exercise for PwD.

Although we selected intervention characteristics (i.e., combined walking and strength exercise, 3 sessions/week for 24 weeks) that previously showed the highest efficacy on physical outcomes in PwD [11, 45], we found no significant effects of exercise on physical function. This was contrary to other successful interventions that showed combined exercise to be related to better endurance, mobility, muscle strength, and balance in PwD [11, 45, 70, 71]. Unfortunately, a previous systematic review on the effects of exercise on physical function in PwD could not yet determine the characteristics of successful interventions [45]. Although a larger training volume in general was related to more improvements in physical function, there is currently not enough evidence to determine how program duration, session duration, frequency, and intensity each contribute to exercise effects on functional outcomes in PwD [5]. Partly, this results from a scarcity of studies that report dose parameters subjectively and/or objectively [5], and a shortage of studies in PwD in which the effects of different exercise doses are compared among randomized subjects or conditions [5], further illustrating the need for the current study. Last, the generally low number of participants (mean *N* = 56) across the aforementioned exercise studies [11, 45, 70, 71] may complicate the interpretation of exercise effects on physical function in PwD. Several other factors may have weakened the exercise effects in our study. First, exercise interventions specifically in daycare or residential care settings have generated conflicting results [72–75]. Perhaps, exercise effects are lower for PwD in daycare or residential care due to stressors related to disease progression, disease awareness, caregiver burden, and irregularity of daily life. Future studies could consider the impact of the living environment on the effects of exercise in PwD. Furthermore, physical function levels of our control group remained stable over the course of the study, which could attest to a confounding effect of daycare or residential care activities. However, this is unlikely as we found a drop in the level of physical activity after the intervention (additional measurements using structured questionnaires with formal and informal caregivers, data not shown). Perhaps, the cognitive stimulation of the control activities afforded physical function benefits which strengthens the evidence for reverse causality in the relationship between physical and cognitive functions that were previously found for gait speed [76]. Furthermore, the flexibility exercises of the control group require coordination which may have afforded cognitive benefits.

### Effects of exercise on cognitive function

We found no effects of exercise vs. control activities on cognitive function and no differences between LI (*d* = − 0.03) and HI (*d* = − 0.04) exercises. Earlier evidence for the effects of exercise on cognition is conflicted for PwD in nursing homes [10, 11, 13, 71, 77] as well as community settings [14, 78–81]. Studies specifically in daycare or residential care settings are scarce. One RCT in PwD attending daycare showed that aerobic training had favorable effects on psychomotor speed only [82]. Altogether, there is a lack of convincing evidence for the efficacy of exercise for cognition in PwD. Shared study characteristics of ten exercise interventions that showed positive effects on cognitive function in PwD [11, 71, 78, 81, 83–88] are a low number of participants (*N* ~ 30 for 9 interventions); lower baseline MMSE scores (MMSE indicative of low-to-moderate dementia for 6 interventions); institutionalized setting (7 interventions performed in NH PwD); program duration between 9 and 24 weeks (9 interventions); multimodal intervention consisting of aerobic training (7 interventions) with added strength, balance, or cognitive training (5 interventions); and high-to-excellent participant attendance and a non-active control group (9 interventions). However, it should be noted that studies with low *N* effect sizes may be overestimated [89]. Furthermore, a previous systematic review in PwD found the reliability of six motor tests for endurance, gait speed, balance, strength, and functional mobility to be good-to-excellent in PwD [90], but the reliability was lower for lower-functioning individuals. Also, dementia-related fluctuations in cognitive function may lower the reliability of cognitive tests in general. Thus, the potential lower reliability of assessments in PwD warrants caution in interpreting the abovementioned and current exercise effects. As mentioned previously for physical function, other factors may weaken the exercise effects on cognitive function. Dementia-related factors such as disease progression, environmental factors, and caregiver burden may weaken the effects of exercise on cognition in PwD. Alternatively, a lack of convincing effects of exercise on cognition may indicate that exercise alone does not sufficiently stimulate cognition in PwD. Diversity in symptoms and disease etiology may require diverse interventions, and exercise could be one option for PwD in addition to cognitive training, social stimulation, and sensory enrichment, preferably as part of a multicomponent program [91, 92]. Recent conceptual models suggest that it may be necessary to perform cognitive and motor tasks in combination and concurrently to increase the efficacy of exercise interventions [93]. The optimal duration for such a multicomponent program is yet to be determined by future studies. In addition to a multicomponent program, a more individualized approach as opposed to a standardized program may be necessary for optimal results [94]. Contrary to the clinical expectations, both the exercise and control participants remained stable over 24 weeks which attests to the beneficial effects of attention and control activities on cognition. Controls participated in recreational activities which may stimulate aspects of cognition in PwD [95]. Indeed, the average MMSE decline of − 0.7 in the control group (Table 3) is lower than the ~ 1.2–4 point decline that was previously found in comparable samples of PwD [80, 96]. To conclude, for PwD, performing activities of any kind may be beneficial for cognition.

Contrary to our expectations, there were no differential effects of the LI and HI phases. We expected a dose-response relationship for intensity between exercise and cognition because higher intensity exercise is related to better fitness parameters [44] which could translate to changes in cognitive function. In a previous meta-analysis, we could not relate exercise intensity to changes in cognitive function in older adults with cognitive impairments [5], but studies that compared exercise intensities among randomized subjects were lacking. This is the first such study in patients with dementia. With this study, we cannot provide evidence that the effects of exercise on cognition can be enhanced by increasing exercise intensity. It should be noted that the distinction LI-HI could be made for strength training, but not convincing for walking. Future studies could investigate whether exercise intensity is related to changes in physiological parameters that may underlie cognitive changes in patients with dementia.

### ApoE4 moderation

We found no significant three-way Group*Time*Carrier interactions for any of the physical or cognitive measures. However, there was a trend for improved MMSE scores for non-carriers in the exercise group and decreased for all other groups (*F*(1,65) = 3.28, *p* = 0.075, Additional file 1: Appendix 4d). This finding complements post hoc findings from the FAB study that showed a significantly better change in global cognition (ADAS-COG) in ApoE4 non-carriers in the exercise group compared to others [97]. A higher rate of clinical decline and atrophy in ApoE4 carriers vs. non-carriers [98] may negate the beneficial effects of physical activity. However, we urge caution when interpreting this result as we found it for one test only, and it was not significant. Thus, at this time, we cannot conclude that ApoE4 carriership is an important moderator in exercise studies with PwD.

### Strengths and limitations

There were several strengths to this study. Our design allowed us to investigate the differential effects of LI and HI exercise, and we carefully monitored exercise intensity both objectively and subjectively. As compared to a three-group design with LI exercise vs. HI exercise vs. control, our current two-group exercise vs. control design ensured that participants could gradually build up exercise intensity and heterogeneity remained as low as possible. Furthermore, we conducted our study in a practical health care setting to strengthen the ecological validity of our findings. Last, we opted for individually supervised sessions in a carefully controlled design.

Several limitations warrant caution in the interpretation of our results. The current results have to be interpreted in light of the limited sample size, further reduced by dropouts. In addition, the large heterogeneity in dementia diagnoses and the large number of unspecified diagnoses may have inflated variation, thereby reducing the power of the current study. Also, in our study, we determined the exercise intensity using the RPE and HRmax as complementary measures, in addition to other variables including progression and variation in exercises, in accordance with the suggestions for determining the dosage of exercise [99]. These methods are not without limitations. HRmax is highly variable in old adults [100] which warrants caution in applying measures of HRmax in exercise studies with old adults. However, we chose to predict HRmax using age as we doubt the feasibility of exercise stress testing in PwD. Subjectively, intensity was determined through observer-rated RPE using a Borg scale, but the Borg scale is not validated in PwD. Furthermore, there may be discrepancies between HRmax and RPE, potentially influenced by beta blocker use or other intrapersonal characteristics. In our study, we instructed the research assistants to rely upon RPE in case of discrepancies. However, this method may have led to some participants being slowed down or stimulated to a larger extent than what was targeted. Furthermore, we expected that potential beneficial exercise effects would become less pronounced over the course of the study, but the current design (12 weeks LI vs. 12 weeks HI exercise) does not allow for confirmation of this expectation. Furthermore, the study was set in fall/winter for logistical reasons, and we cannot rule out seasonal influences on dementia decline. Unfortunately, we have no information on the neurobiological factors (i.e., changes in IGF-1, VEGF, BDNF levels) hypothesized to underlie the beneficial effect of exercise on brain health. Such information is important because Alzheimer’s disease (AD) has been associated with lower serum levels of IGF-1 [101] and BDNF [102]. Although lower levels in these neurobiological factors could leave more room for improvement, it is also possible that the neurobiological system is less responsive in PwD [103, 104]. It is left to future exercise studies to account for changes in such neurobiological factors in PwD. Last, the cognitive tests that we employed are often used but not all psychometrically evaluated in PwD [50], and dementia-related fluctuations in cognitive function may lower the reliability of cognitive tests in general. Future studies are needed to validate commonly used neuropsychological tests and adapt tests to suit the needs of PwD.

## Conclusions

Exercise was superior to control activities for better gait speed. This is an important result because gait speed has high clinical relevance in older adults. There was a dose-response relationship for the intensity between exercise and gait speed improvements, which may have been fueled by strength improvements in the HI phase. We found no significant effects of exercise on the other physical functions. Exercise was not superior to control activities for cognition in PwD. With gait speed as an exception, we found no evidence that higher intensity exercise afforded more physical or cognitive benefits. Altogether, our results are not in contrast with the recommendation for physical activity over control activities for PwD, preferably at higher intensities, in accordance with ACSM’s guidelines [46]. The current results should be carefully interpreted in light of the limited sample size (*N* = 91 PwD included with *N* = 118 inclusions necessary for sufficient power), further reduced by selective dropout. Although our overall dropout rate of 24% was as expected, selective dropout in particular may be prevented in future studies with a more gradual increase in exercise session duration or frequency (for lower-functioning PwD in an exercise group) or more challenging control activities (for higher-functioning PwD in a control group). With an eye on future studies, diversity in dementia symptoms and etiology may require diverse interventions and future studies are needed to investigate whether multicomponent programs including but not limited to physical exercise, cognitive training, social stimulation, and sensory enrichment are indeed more effective for physical and cognitive function in PwD. In addition to multicomponent programs, a more individualized approach as opposed to standardized programs may be necessary for optimal results. For personalization of treatments, we advise future researchers to collect data on the characteristics of responders and non-responders to lifestyle programs including, but not limited to, physical exercise.

## Supplementary information

**Additional file 1: Appendix 1.** Adaptation of sets and repetitions during strength sessions. **Appendix 2a.** Description of the physical function tests. **Appendix 2b.** Description of the cognitive function tests. **Appendix 3.** Training characteristics for the exercise and control group. **Appendix 4a.** Means and standard deviations for the imputed cognitive test scores for ApoE4 carriers vs. non-carriers. **Appendix 4b.** Means and standard deviations for the imputed physical test scores for ApoE4 carriers vs. non-carriers. **Appendix 4c.** Three-way time x group x ApoE4 carriership analyses for cognitive and physical functions. **Appendix 4d.** Three-way interaction (Time*Group*Carrier) for MMSE score. **Appendix 5.** Means and standard deviations of the follow-up data for gait speed, leg strength and STROOP.

## Data Availability

The datasets used and/or analyzed during the current study are available from the corresponding author on reasonable request.

## Abbreviations

6MWS: 6-Meter walking speed
6MWT: 6-Minute walk test
ACSM: American College of Sports Medicine
AD: Alzheimer’s disease
ANCOVA: Analysis of covariance
ApoE4: Apolipoprotein-E4
BDNF: Brain-derived neurotrophic factor
CI: Confidence interval
DBI: Drug Burden Index
DSBW: Digit Span Backward
DSFW: Digit Span Forward
ES: Effect size
FCI-18: Functional Comorbidity Index-18
Fluency: Phonemic fluency test
HI: High-intensity
HRmax: Maximum heart rate
IGF-1: Insulin-like growth factor-type I
LI: Low-intensity
MMSE: Mini-Mental State Examination
PwD: Patients with dementia
RPE: Rate of perceived exertion
SE: Standard error
SPPB: Short Physical Performance Battery
TMTA: Trail Making Test A
TUG: Timed Up & Go
VEGF: Vascular endothelial growth factor
VMSBW: Visual Memory Span Backward
VMSFW: Visual Memory Span Forward
