# Supplementary material for: Effects of low- and high-intensity physical exercise on physical and cognitive function in older persons with dementia: a randomized controlled trial
Source: Alzheimers Res Ther. 2020 Mar 19;12:28. doi: 10.1186/s13195-020-00597-3 (PMC7082953; doi:10.1186/s13195-020-00597-3)
Supplement: Supplementary file 1 — Additional file 1: Appendix 1. Adaptation of sets and repetitions during strength sessions. Appendix 2a. Description of the physical function tests. Appendix 2b. Description of the cognitive function tests. Appendix 3. Training characteristics for the exercise and control group. Appendix 4a. Means and standard deviations for the imputed cognitive test scores for ApoE4 carriers vs. non-carriers. Appendix 4b. Means and standard deviations for the imputed physical test scores for ApoE4 carriers vs. non-carriers. Appendix 4c. Three-way time x group x ApoE4 carriership analyses for cognitive and physical functions. Appendix 4d. Three-way interaction (Time*Group*Carrier) for MMSE score. Appendix 5. Means and standard deviations of the follow-up data for gait speed, leg strength and STROOP. [file 13195_2020_597_MOESM1_ESM.docx]

**Appendix 1. Adaptation of sets and repetitions during strength sessions.**

Each participant started every exercise with 2 sets of 6 repetitions without added weights. When the participant had yet to reach the target intensity, another 2 repetitions were added to a maximum of 12 repetitions. Further increasing of intensity was done by adding an extra set, with a total of 3 sets of 12 repetitions per exercise. If it was necessary to decrease intensity, 2 repetitions were subtracted to a minimum of 2 sets of 2 repetitions per exercise. The participant started the strength session thereafter with the number of sets and repetitions that he/she successfully completed during the previous session. In the HI phase every participant started with 2 sets of 6 repetitions with an ankle weight of 0.5 kg. The method of increasing and decreasing the intensity was equivalent to the LI phase, but after 3 sets of 12 repetitions the participants started at 2 sets of 8 repetitions with another 0.5 kg ankle weight and so forth.

**Appendix 2a. Description of the physical function tests.**

The Six-Meter Walk Test (6MWT) measures endurance. Participants walk as many rounds as possible of two cones set 10m apart within 6 minutes. The total distance walked is recorded. The SPPB assesses lower body strength and functional mobility. The Short Physical Performance Battery (SPPB) includes standing balance (feet together, semi-tandem, tandem, one-leg stance), habitual 6-meter walking speed (6MWS, m/s) and 5 times chair stand (5STS). A score of 0 (lowest) to 4 (highest) is given per condition so that the total score of the SPPB lies between 0-12. The FICSIT-4 measures static balance (feet together, semi-tandem, tandem, one-leg stance). Participants have to hold each stance for 10s. Scores range between 0 (no stances performed ≥10s) to 5 (all stances performed ≥10s). We assessed lower body muscle strength with the Quadriso table, which we based on the Quadrisotester of Verkerke et al. [1]. The Quadriso table was found to be a feasible, reliable and valid measure of maximal voluntary isometric force of the quadriceps muscle (unpublished data). Participants are instructed to sit on a table with a force measuring device above the ankle. Participants have to generate maximal force for 3s. Three trials are performed for each leg and the maximum force in Newton (N) is recorded. Last, the TUG measures functional mobility. Participants are instructed to rise from a chair, walk 3 meters and sit down again. We used the fastest time of two trials as outcome.

**References**

[1] Verkerke GJ, Lemmink KA, Slagers AJ, Westhoff MH, van Riet GA, Rakhorst G. Precision, comfort and mechanical performance of the Quadriso-tester, a quadriceps force measuring device. Med Biol Eng Comput 2003, 41:283-289.

**Appendix 2b. Description of the cognitive function tests.**

Global cognition was assessed with the Mini Mental State Examination. Scores range from 0-30 with 30 being the best performance. The Trail Making Test A (TMTA) measures psychomotor speed. Participants have to sequentially connect numbers 1-25. We recorded the time to complete the test (s) with 240s as cut-off score. The Digit Span Forward measures verbal memory span. Participants repeat a sequence of digits of increasing length. In the Digit Span Backward, a measure of verbal working memory, participants have to repeat the sequences of digits in reverse order. For both tests, the number of correct responses is used as outcome measure. The Visual Memory Span Forward and Backward (VMSFW and VMSBW) are measures of respectively visual memory span and visual working memory. In the VMSFW, participants have to tap a block sequence of increasing length. In the VMSBW, the sequence of blocks has to be tapped in reverse order. The number of correct responses is recorded in both conditions. The STROOP test is used to assess basic attentional processing and inhibitory control. In condition I, participants read the names of four colors (blue, red, yellow, green). In condition II participants are asked to name the four colors. Condition III is the interference condition, in which participants have to name the color of words that are printed in incongruent colors (i.e. the word ‘blue’ printed in red ink). In all conditions, we recorded the number of correct responses in 45s. An interference score is obtained by dividing the scores on condition II by condition III. Larger scores represent more interference. The Phonemic Fluency Test (Fluency) was used as executive function measure. Participants need to name as many words as possible that start with a given letter within 1 minute. The number of correct responses in three attempts is recorded.

**Appendix 3. Training characteristics for the exercise and control group**

| Characteristic | Exercise (N=39) | | | | | | Control (N=30) | Exercise vs. control |
| --- | --- | --- | --- | --- | --- | --- | --- | --- |
|  | Walking | |  | Strength | |  |  |  |
|  | LI phase^a^ | HI phase^a^ | Difference  HI-LI | LI phase^a^ | HI phase^a^ | Difference  HI-LI |  |  |
| Adherence  (% performed sessions/offered sessions, mean, SD) | 57.3 (23.4) | 58.0 (29.2) | 0.71 (14.2) | 62.4 (23.1) | 65.1 (29.8) | 2.70 (20.5) | 69.5 (18.5) | *U*=475^h^ |
| Heart rate, beats/min^-1b^ (mean, SD) | 94.7 (12.1) | 96.2 (12.4) | 1.41 (8.51) | n\a | n\a | n\a | 74.6 (11.4) | t(60)=-7.18**^i^ |
| Heart rate difference after-before session, beats/min^-1b^ (mean, SD) | 18.7 (12.4) | 19.0 (11.8) | 0.32 (15.2) | n\a | n\a | n\a | -0.52 (2.92) | *U*=986**^i^ |
| Maximum heart rate, beats/min^-1c^ (SD) | 132.9 (21.2) | 137.8 (19.8) | 4.88 (19.2) | n/a | n/a | n/a | n/a | n/a |
| RPE^d^ (mean, SD) | 9.10 (1.25) | 12.2 (1.73) | 3.09 (1.58)** | 8.95 (1.35) | 12.6 (2.15) | 3.61 (1.99)** | 7.03 (1.16) | *U*=992**^j^ |
| RPE difference after-before session^e^ (mean, SD) | 2.78 (1.44) | 6.03 (2.49) | 3.26 (2.12)** | n\a | n\a | n\a | 0.06 (0.28) | *U*=1134**^k^ |
| Distance walked, km^f^ (mean, SD) | 1.30 (0.55) | 1.34 (0.62) | 0.04 (0.28) | n\a | n\a | n\a | n\a | n\a |
| Number of repetitions^g^ (mean, SD) | n\a | n\a | n\a | 78.5 (46.6) | 107.6 (75.9) | 29.1 (63.5)** | n\a | n\a |
| Added weight, kg^g^ (mean, SD) | n\a | n\a | n\a | n\a | 0.71 (0.43) | n\a | n\a | n\a |
| Quality^g^ (mean, SD) | n\a | n\a | n\a | 2.55 (0.41) | 2.58 (0.50) | 0.03 (0.44) | n\a | n\a |
| Participant satisfaction^d^ (mean, SD) | 1.82 (0.23) | 1.63 (0.37) | -0.19 (0.28)** | 1.80 (0.23) | 1.71 (0.41) | -0.09 (0.35) | 1.83 (0.25) | *U*=334*^h^ |

^a^LI = low intensity phase; HI = high intensity phase. n\a = not applicable. *p<0.05; **p<0.01; †p<0.1. ^b^N=1 upper outlier removed, N=5 missing for LI and/or HI walking, N=1 missing for control; ^c^N=2 upper outlier removed, N=5 missing for LI and/or HI walking, ^d^N=5 missing for LI and/or HI walking, N=4 missing for LI and/or HI strength;  ^e^N=5 missing for LI and/or HI walking; ^f^N=6 missing for exercise group; ^g^N=4 missing for LI and/or HI strength. ^h^Average total exercise vs. control; ^i^average walking LI/HI vs. control; ^j^average walking LI + strength LI vs. control; ^k^walking LI vs. control.

**Appendix 4a. Means and standard deviations for the imputed cognitive test scores for ApoE4 carriers vs. non-carriers.**

|  |  | Exercise | | | ES^c^ [95% CI] Baseline – 24 wks | Control | | | ES^c^ [95% CI]  Baseline – 24 wks |
| --- | --- | --- | --- | --- | --- | --- | --- | --- | --- |
| Test^a^ | Group | Baseline | 12 weeks | 24 weeks |  | Baseline | 12 weeks | 24 weeks |  |
| MMSE (score) | Carriers | 21.1 (3.61) | 20.0 (3.31) | 18.6 (4.54) | 0.66 [0.00, 1.32] | 21.1 (4.19) | 21.2 (4.69) | 20.3 (4.94) | -0.01 [-0.75, 0.73] |
|  | Non-carriers | 21.6 (4.28) | 21.8 (5.07) | 22.0 (4.50) |  | 18.5 (4.96) | 18.2 (6.01) | 17.7 (6.33) |  |
| TMTA (seconds) | Carriers | 127 (62.2) | 143 (62.7) | 139 (60.1) | 0.19 [-0.46, 0.84] | 150 (72.4) | 148 (57.8) | 142 (54.8) | -0.14 [-0.88, 0.61] |
|  | Non-carriers | 116 (66.9) | 105 (60.8) | 116 (69.1) |  | 160 (61.8) | 161 (64.3) | 160 (58.0) |  |
| STROOP word (#correct responses) | Carriers | 48.3 (22.5) | 52.0 (17.0) | 46.4 (17.7) | 0.11 [-0.54, 0.75] | 56.7 (20.6) | 55.3 (18.8) | 51.2 (21.4) | 0.16 [-0.58, 0.90] |
|  | Non-carriers | 59.4 (18.9) | 58.2 (21.6) | 59.6 (20.8) |  | 47.0 (23.5) | 47.3 (23.9) | 44.9 (19.3) |  |
| STROOP colour  (#correct responses) | Carriers | 36.9 (13.8) | 38.2 (15.5) | 37.3 (14.3) | 0.21 [-0.44, 0.85] | 37.9 (17.5) | 39.5 (18.1) | 41.4 (15.1) | -0.19 [-0.94, 0.55] |
|  | Non-carriers | 45.0 (14.9) | 48.8 (17.1) | 48.4 (16.6) |  | 35.4 (18.1) | 34.1 (18.2) | 35.7 (15.6) |  |
| STROOP colour-word  (#correct responses) | Carriers | 18.2 (9.71) | 14.8 (8.91) | 13.7 (8.64) | 0.73 [0.06, 1.40] | 14.9 (8.79) | 15.5 (9.36) | 18.0 (8.24) | 0.02 [-0.72, 0.76] |
|  | Non-carriers | 17.1 (11.1) | 18.0 (12.2) | 20.2 (11.5) |  | 12.6 (9.34) | 14.4 (8.32) | 15.9 (9.33) |  |
| STROOP interference  quotient^b^ | Carriers | 3.31 (3.82) | 4.09 (4.07) | 4.48 (3.78) | 0.66 [0.00, 1.33] | 3.43 (2.81) | 3.18 (1.91) | 2.79 (1.49) | 0.28 [-0.48, 1.04] |
|  | Non-carriers | 3.58 (2.61) | 4.00 (3.35) | 2.78 (1.10) |  | 4.72 (4.70) | 3.11 (2.47) | 3.16 (2.60) |  |
| DSFW  (#correct responses) | Carriers | 6.67 (1.88) | 6.80 (2.04) | 6.56 (1.77) | -0.02 [-0.66, 0.63] | 6.83 (1.53) | 6.92 (1.78) | 6.83 (1.75) | 0.10 [-0.64, 0.84] |
|  | Non-carriers | 6.90 (1.70) | 7.38 (2.23) | 6.76 (1.88) |  | 6.33 (1.57) | 6.36 (1.59) | 6.51 (2.04) |  |
| DSBW  (#correct responses) | Carriers | 3.94 (1.51) | 3.78 (1.34) | 3.79 (1.34) | 0.21 [-0.43, 0.86] | 4.08 (1.38) | 4.50 (1.38) | 3.92 (1.73) | 0.03 [-0.71, 0.78] |
|  | Non-carriers | 4.05 (1.32) | 4.19 (1.71) | 4.19 (1.30) |  | 4.23 (1.32) | 4.01 (1.55) | 4.12 (1.54) |  |
| VMSFW  (#correct responses) | Carriers | 5.70 (2.18) | 5.25 (1.54) | 5.21 (1.67) | -0.06 [-0.71, 0.58] | 5.17 (2.12) | 4.75 (2.26) | 4.33 (1.97) | 0.32 [-0.43, 1.06] |
|  | Non-carriers | 5.85 (1.78) | 5.71 (1.62) | 5.25 (1.41) |  | 4.83 (1.59) | 4.51 (1.52) | 4.61 (2.14) |  |
| VMSBW  (#correct responses) | Carriers | 3.72 (1.96) | 3.94 (1.99) | 4.34 (1.71) | -0.36 [-1.01, 0.29] | 4.75 (1.98) | 4.67 (2.15) | 4.08 (2.15) | 0.26 [-0.49, 1.00] |
|  | Non-carriers | 4.81 (2.04) | 4.49 (1.87) | 4.75 (1.74) |  | 3.99 (1.75) | 3.46 (1.68) | 3.82 (1.90) |  |
| Fluency  (#correct responses) | Carriers | 18.4 (8.76) | 18.1 (8.60) | 21.6 (8.31) | -0.11 [-0.76, 0.53] | 16.1 (8.19) | 16.8 (10.2) | 19.1 (11.6) | -0.26 [-1.00, 0.49] |
|  | Non-carriers | 19.3 (6.93) | 18.4 (8.20) | 21.6 (8.86) |  | 13.8 (10.2) | 13.2 (9.79) | 14.4 (7.67) |  |

ApoE4 = Apolipoprotein e4. NTotal=69; Exercise group: N=18 carriers vs. N=21 non-carriers; Control group: N=12 carriers vs. N=18 non-carriers. ^a^MMSE = Mini-Mental State Examination; TMTA = Trail Making Test A; DSFW = Digit Span Forward; DSBW = Digit Span Backward; VMSFW = Visual Memory Span Forward; VMSBW = Visual Memory Span Backward; Fluency = Phonemic fluency. ^b^Colour/colour-word score; N=1 missing for control. ^c^ES = Effect size; positive effect sizes are in favor of non-carriers. There were no significant baseline differences (all p>0.05).

**Appendix 4b. Means and standard deviations for the imputed physical test scores for ApoE4 carriers vs. non-carriers.**

|  | Group | Exercise | | | ES^b^ [95% CI]  Baseline – 24 wks | Control | | | ES^b^ [95% CI] Baseline – 24 wks |
| --- | --- | --- | --- | --- | --- | --- | --- | --- | --- |
| Test^a^ |  | Baseline | 12 weeks | 24 weeks |  | Baseline | 12 weeks | 24 weeks |  |
| 6MWT (m) | Carriers | 292 (94.1) | 288 (91.6) | 296 (102) | 0.15 [-0.49, 0.80] | 257 (93.9) | 221 (92.2) | 249 (89.6) | 0.24 [-0.51, 0.98] |
|  | Non-carriers | 264 (85.3) | 274 (84.6) | 282 (91.0) |  | 219 (84.0) | 224 (106) | 231 (87.6) |  |
| SPPB (score) | Carriers | 8.45 (2.24) | 9.12 (2.34) | 9.06 (1.99) | -0.33 [-0.98, 0.32] | 7.75 (2.34) | 7.58 (2.06) | 7.67 (1.56) | -0.06 [-0.80, 0.69] |
|  | Non-carriers | 9.01 (2.21) | 9.25 (2.48) | 8.87 (2.64) |  | 7.78 (1.99) | 7.58 (2.24) | 7.57 (2.86) |  |
| 6MWS (m/s) | Carriers | 0.97 (0.30) | 0.95 (0.27) | 1.02 (0.29) | -0.02 [-0.67, 0.62] | 0.88 (0.19) | 0.85 (0.23) | 0.84 (0.21) | 0.19 [-0.57, 0.94] |
|  | Non-carriers | 0.90 (0.33) | 0.91 (0.25) | 0.95 (0.23) |  | 0.84 (0.21) | 0.83 (0.25) | 0.76 (0.30) |  |
| FICSIT-4 (score) | Carriers | 3.31 (1.27) | 3.50 (1.06) | 3.36 (1.41) | -0.20 [-0.84, 0.45] | 3.13 (1.57) | 2.92 (1.36) | 3.08 (1.00) | 0.21 [-0.55, 0.97] |
|  | Non-carriers | 3.41 (0.92) | 3.41 (1.33) | 3.22 (1.29) |  | 2.74 (1.26) | 2.73 (1.20) | 2.99 (1.61) |  |
| TUG (s) | Carriers | 13.0 (5.49) | 13.2 (5.58) | 13.3 (6.33) | 0.20 [-0.45, 0.84] | 15.4 (5.31) | 15.6 (5.47) | 15.9 (5.57) | -0.06 [-0.83, 0.71] |
|  | Non-carriers | 15.7 (6.72) | 13.9 (5.64) | 14.7 (6.94) |  | 18.5 (5.52) | 19.3 (8.53) | 19.3 (7.92) |  |
| Leg strength (N) | Carriers | 181 (80.1) | 197 (80.4) | 206 (82.9) | -0.25 [-0.90, 0.40] | 184 (64.4) | 199 (64.2) | 208 (86.1) | -0.50 [-1.25, 0.25] |
|  | Non-carriers | 220 (98.5) | 217 (113) | 221 (107) |  | 191 (42.5) | 163 (51.1) | 185 (51.3) |  |

ApoE4 = Apolipoprotein e4. NTotal=69; Exercise group: N=18 carriers vs. N=21 non-carriers; Control group: N=12 carriers vs. N=18 non-carriers.. ^a^6MWT = Six Meter Walk Test; SPPB = Short Physical Performance Battery; 6MWS = 6 meter walk speed; TUG = Timed Up&Go. ^b^ES = Effect size; positive effect sizes are in favor of non-carriers. There were no significant baseline differences (all p>0.05).

**Appendix 4c. Three-way time x group x ApoE4 carriership analyses for cognitive and physical functions**

| **Domain** | **Test^a^** | **F(1,65)^c^, p** |
| --- | --- | --- |
| Cognition | MMSE (score) | 3.28, p=0.075† |
|  | TMTA (seconds) | 0.69, p>0.05 |
|  | STROOP word (#correct responses) | 0.03, p>0.05 |
|  | STROOP colour (#correct responses) | 1.28, p>0.05 |
|  | STROOP colour-word (#correct responses) | 2.56, p>0.05 |
|  | STROOP interference quotient^b^ | 0.87, p>0.05 |
|  | DSFW (#correct responses) | 0.21, p>0.05 |
|  | DSBW (#correct responses) | 0.24, p>0.05 |
|  | VMSFW (#correct responses) | 0.65, p>0.05 |
|  | VMSBW (#correct responses) | 1.70, p>0.05 |
|  | Fluency (#correct responses) | 0.23, p>0.05 |
| Physical function | 6MWT (m) | 0.04, p>0.05 |
|  | SPPB (score) | 0.44, p>0.05 |
|  | 6MWS (m/s) | 1.02, p>0.05 |
|  | FICSIT-4 (score) | 0.93, p>0.05 |
|  | TUG (s) | 0.29, p>0.05 |
|  | Leg strength (N) | 0.06, p>0.05 |

^a^MMSE = Mini-Mental State Examination; TMTA = Trail Making Test A; DSFW = Digit Span Forward; DSBW = Digit Span Backward; VMSFW = Visual Memory Span Forward; VMSBW = Visual Memory Span Backward; Fluency = Phonemic fluency; 6MWT = Six Meter Walk Test; SPPB = Short Physical Performance Battery; 6MWS = 6 meter walk speed; TUG = Timed Up&Go. ^b^Colour/colour-word score. ^c^Repeated Measures ANOVA with time (pre- vs. postteset) as within-subjects factor, and group (exercise vs. control) and ApoE4 carrier (carrier vs. non-carrier) as between-subjects factors; three-way interaction. †significant at p<0.1.

**Appendix 4d. Three-way interaction (Time*Group*Carrier) for MMSE score.
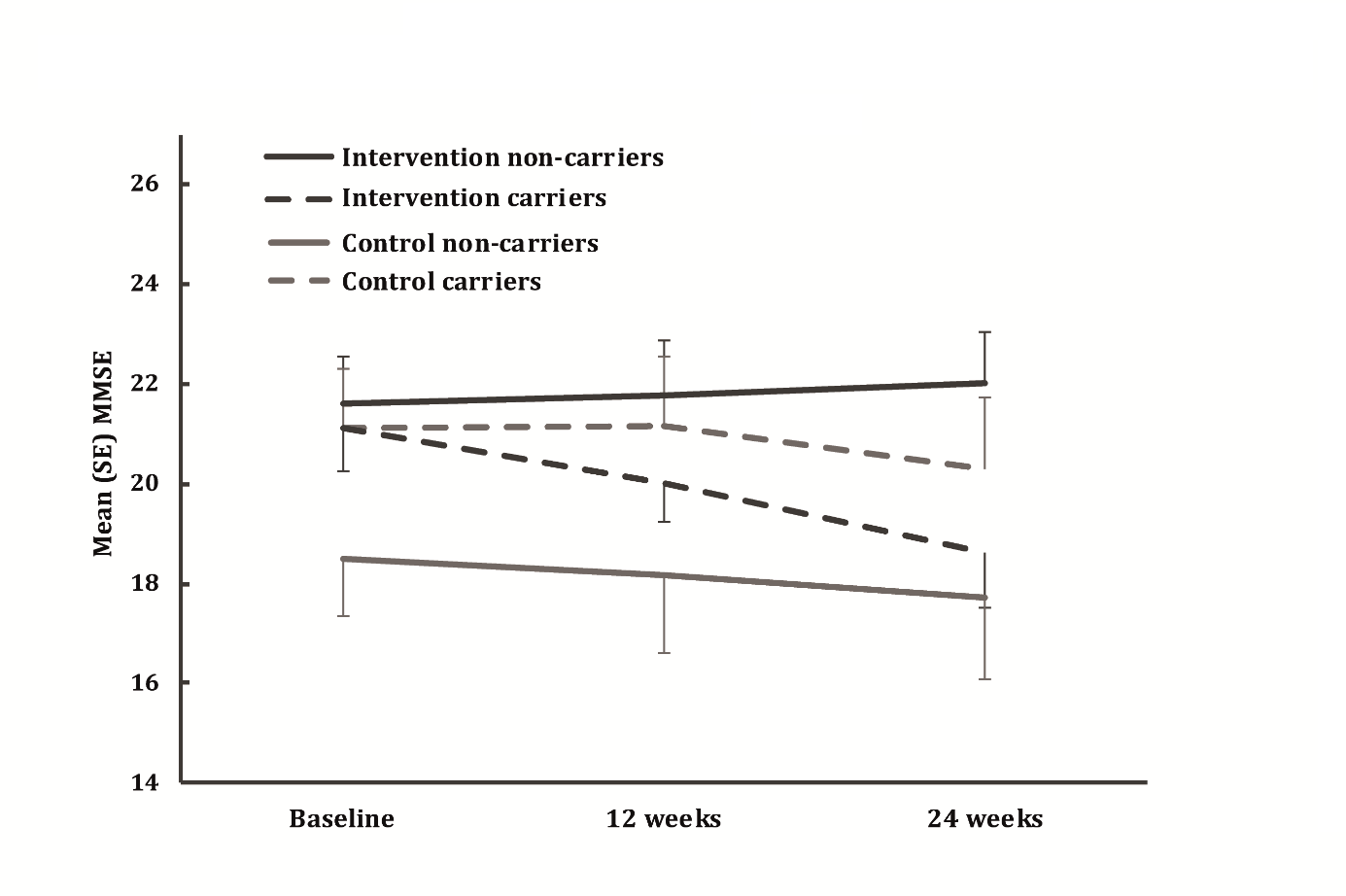
Appendix 5. Means and standard deviations of the follow-up data for gait speed, leg strength and STROOP**

|  |  |  |
| --- | --- | --- |
| Test^a^ | Group | 36 weeks follow-up |
| STROOP word (#correct responses) | Exercise | 56.0 (21.2) |
|  | Control | 47.3 (19.3) |
| STROOP colour (#correct responses) | Exercise | 43.9 (17.2) |
|  | Control | 37.2 (15.5) |
| STROOP colour-word (#correct responses) | Exercise | 17.3 (11.4) |
|  | Control | 13.1 (8.87) |
| STROOP interference quotient^a^ | Exercise | 3.67 (3.00) |
|  | Control | 4.50 (4.31) |
| 6MWS^b^ (m/s) | Exercise | 0.89 (0.25) |
|  | Control | 0.73 (0.22) |
| Leg strength (N) | Exercise | 195 (78.1) |
|  | Control | 172 (56.9) |

^a^Colour/colour-word score. ^b^6MWS = 6 meter walk speed.
